# Knockdown of long non-coding RNA PVT1 induces apoptosis of fibroblast-like synoviocytes through modulating miR-543-dependent SCUBE2 in rheumatoid arthritis

**DOI:** 10.1186/s13018-020-01641-6

**Published:** 2020-04-15

**Authors:** Junxia Wang, Xianghui Kong, Haijian Hu, Shunfang Shi

**Affiliations:** Department of Rheumatism, Linyi Central Hospital, No. 17, Jiankang Road, Yishui Town, Linyi, 276400 Shandong People’s Republic of China

**Keywords:** Long non-coding RNA PVT1, MicroRNA-543, SCUBE2, Rheumatoid arthritis, Synovial cells, Interleukin-1β

## Abstract

**Background:**

Rheumatoid arthritis (RA), a kind of autoimmune disorder, is featured by many physical symptoms and proliferation of fibroblast-like synoviocytes (FLSs). The relevance of long non-coding RNAs (lncRNAs) in the progression of RA has been probed. Hence, the goal of this report was to investigate the action of plasmacytoma variant translocation 1 (PVT1), a lncRNA, in FLSs and the basic mechanism.

**Methods:**

Initially, RA rats were developed to evaluate the expression of PVT1, microRNA-543 (miR-543), and signal peptide-CUB-EGF-like containing protein 2 (SCUBE2) in synovial tissues. Enhancement or loss of PVT1 or miR-543 was achieved to explore their effects on proliferation, cell cycle, and apoptosis of FLSs. The interaction between PVT1 and miR-543 and between miR-543 and its putative target SCUBE2 was examined to elucidate the correlations. Finally, the protein expression of proliferation- and apoptosis-associated genes were assessed by western blot assays.

**Results:**

PVT1 was overexpressed in synovial tissues from RA patients through microarray expression profiles. The PVT1 and SCUBE2 expression was boosted, and miR-543 was reduced in synovial tissues of rats with RA. PVT1 specifically bound to miR-543, and miR-543 negatively regulated SCUBE2 expression. Overexpression of PVT1 or silencing of miR-543 enhanced SCUBE2 expression, thereby promoting proliferation and interleukin-1β (IL-1β) secretion, while inhibiting apoptosis rate of FLSs. Conversely, si-SCUBE2 reversed the role of miR-543 inhibitor.

**Conclusion:**

The key findings support that PVT1 knockdown has the potency to hinder RA progression by inhibiting SCUBE2 expression to sponge miR-543.

## Background

Rheumatoid arthritis (RA) is a type of chronic disorder linked to inflammation and autoimmune which principally disturbs the joints and may contribute to accumulating joint injuries and irreversible disability if inadequately treated [1]. Current advance in outcomes has been achieved as a consequence of a more thorough comprehension of RA pathophysiology and progresses in improved outcome measures and therapies [2]. In RA, fibroblast-like synoviocytes (FLSs), one of the most common cell types on the pannus-cartilage junction, might culminate in joint destruction via the generation of cytokines, chemokines, and matrix-degrading molecules [3]. Moreover, after obtaining an aggressive phenotype, FLSs will become resistance to apoptosis and show elevated migration and invasion abilities to surrounding tissues, such as the bone and cartilage, thus promoting angiogenesis, cell growth, and activation of immune cells [4]. Hence, clarifying the molecular mechanism in which FLSs function as pathological factors of RA might cope with the urgency in the treatment of RA [5].

Recently, long non-coding RNAs (lncRNAs), a set of non-coding RNAs longer than 200 nucleotides, have been implicated in multiple functional changes and numerous dysregulated cells in RA patients, including FLSs [6]. Plasmacytoma variant translocation 1 (PVT1) has been extensively investigated in cancers, including lung cancer [7], colorectal cancer [8], and ovarian cancer [9] where PVT1 was promoted and facilitated cell proliferation and invasion. Besides, the knockdown of PVT1 has been lately reported to suppress inflammation of FLSs and to induce apoptosis in RA through demethylation of sirtuin 6 [10]. lncRNAs have been revealed to modulate the expression of microRNAs (miRNAs) through the competitive endogenous RNA (ceRNA) mechanism, thereby participated in the development of different kinds of autoimmune diseases, including RA [11, 12]. MiR-543-3p has been verified to exert a protective role over neurons through reducing inflammatory response in spinal cord injury [13]. Also, signal peptide-CUB-epidermal growth factor-like containing protein (SCUBE) was highly expressed in the majority of vascularized tissues and primary osteoblasts and bones and could be detected in inflammation- and hypoxia-related disease conditions [14]. A miRNA chromosome 19 miRNA cluster was found to target SCUBE2 to culminate in phenotype of triple-negative breast cancers [15]. In this study, SCUBE2 was observed to be a putative target gene of miR-543, which might interact with PVT1 in RA-FLSs. On the basis of the above literature research, we conjectured that PVT1 might influence the roles of RA-FLSs through regulating miR-543-mediated SCUBE2. Henceforth, our intention was to examine the role of PVT1 on the proliferation, interleukin-1β (IL-1β) release, and apoptosis in RA-FLSs to illuminate the underlying mechanism contributed to RA progression.

## Methods

### Microarray preparation

In short, synovial tissues from 3 healthy people and 3 patients with RA were obtained to extract total RNA, from which 0.5 μg RNA was used to synthesize complementary deoxyribonucleic acid (cDNA) using a GeneChip 3′In Vitro Transcription (IVT) Express Kit (902789, Thermo Fisher Scientific Inc., Waltham, MA, USA). Then, cDNA was segmented and hybridized with human lncRNA expression array V3.0 (AS-LNC-H-V4.0, Arraystar Inc., Rockville, MD, USA). After hybridization, the array was scanned with the GeneChipTM Scanner 3000 7G system (000213, Thermo Fisher Scientific).

### Induction of a RA model in rats

Fourteen healthy Wistar rats (female; age, 6–8 weeks, weight, 140–150 g) were fed at 25–27 °C under 45–50% humidity with free access to food and water. These rats were randomized into a control group (*n* = 7) and a RA group (*n* = 7). According to the previous literature [10], the rats were subjected to 3% pentobarbital sodium injection. Then, the RA rats were administrated with Freund’s complete adjuvant (0.1 mL) at the right posterior toe, and the control rats were administrated with 0.1 mL phosphate-buffered saline (PBS). A digital micrometer was used to measure the size of the posterior feet of each rat, and the swelling degree was calculated by comparing it with the size measured at the baseline.

### Culture and treatment of FLSs in RA

RA-FLSs from American Type Culture Collection (Manassas, VA, USA) were grown at a temperature of 37 °C with 5% CO_2_. When cells were covered with 80% microscopic view, they were detached with trypsin (2.5 g/L) for passage. After being cultured in an incubator for 24 h, plasmids were transfected into RA-FLSs with Lipofectamine 2000 (Thermo Fisher Scientific). After 4 h, the medium was replaced with normal culture medium for further culture, and then the following experiments were carried out. FLSs were transfected with overexpressed (oe)-PVT1, short hairpin RNA- (shRNA; sh)-PVT1, sh-SCUBE2, miR-543 mimic, miR-543 inhibitor + sh-SCUBE2 or miR-543 inhibitor + sh-PVT1 with oe-negative control (NC), sh-NC, mimic NC, inhibitor NC, or miR-543 inhibitor + sh-NC as controls.

### RNA isolation and reverse transcription (RT)-PCR assay

The total RNA was isolated from the tissues using Trizol (16096020, Thermo Fisher Scientific). Totally, 5 μg RNA was then synthesized into complementary deoxyribonucleic acid (cDNA) using a cDNA kit (K1622; Fermentas Inc., Ontario, CA, USA). Subsequently, RT-qPCR is carried out following the protocol of TaqMan Gene Expression Assays (Applied Biosystems, Foster City, CA, USA) with cDNA as a template. U6 and glyceraldehyde-3-phosphate dehydrogenase (GAPDH) were applied as the internal controls for the normalization of miRNA and mRNA, respectively. Obtained results were reproducible in three independent experiments. The primer sequences used are demonstrated in Table 1. The expression of PVT1 and miR-543 and the SCUBE2 mRNA expression were measured by the 2-^ΔΔCt^ method [16].

**Table 1:** RT-qPCR primer sequences

Primer sequence	Forward (5′-3′)	Reverse (5′-3′)
PVT1	GGTACCGAGCTCGGATCCTCAAGATGGCTGTGCC	CGCCACTGTGCTGGATGATAGAAAAAGAATTTAATAG
miR-543	GGAAACATTCGCGGTGC	GTGCGTGTCGTGGAGTCG
SCUBE2	GCAAGTTTGCGTCAACACAG	GACGCCCTTTACTTCCACAC
U6	TGCTCGCTTCGGCAGC	AAAAATATGGAACGCTTCACG
GAPDH	TTGGTATCGTGGAAGGACTCA	TGTCATCATATTTGGCAGGTT

*RT-qPCR* reverse transcription-quantitative polymerase chain reaction, *PVT1* plasmacytoma variant translocation 1, *miR-543* microRNA-543, *SCUBE2* signal peptide-CUB-EGF-like containing protein 2, *GAPDH* glyceraldehyde-3-phosphate dehydrogenase

### Western blot assays

The tissues or cells of each group were ice-bathed for 30 min with the lysis buffer supplemented with phenylmethyl sulfonylfluoride and subjected to a 15-min centrifugation at 10000 rpm at 4 °C. The content of total protein was examined using a bicinchoninic acid kit (Thermo). After being separated by the means of 10% sodium dodecyl sulfate and polyacrylamide gel, the protein sample was transblotted to a polyvinylidene difluoride membrane (Amersham Pharmacia, Piscataway, NJ, USA). Followingly, the membranes were then probed at 4 °C with antibodies against SCUBE2 (ab105378, 1:1000), proliferating cell nuclear antigen (PCNA; ab152112, 1:1000), Ki67 (ab92742, 1:2000), B cell lymphoma/leukemia 2 (Bcl-2; ab194583, 1:1000), Bcl-2-associated X protein (Bax; ab53154, 1:1000), and internal control GAPDH (ab9485, 1:2000) overnight. Then, they were blotted at room temperature for 1 h with the horseradish peroxidase-labeled goat anti-rabbit secondary antibody to IgG (ab6721, 1:2000). All antibodies used were obtained from Abcam Inc. (Cambridge, UK). Finally, immunoreactive bands were detected by an electrochemiluminescence (GE Healthcare, Chicago, IL, USA). We applied Image Pro Plus 6.0 (Media Cybernetics, Rockville, MD, USA) to conduct quantitative analysis.

### 5-Ethynyl-2′-deoxyuridine (EdU) staining

The cells of each group in logarithmic growth phase were seeded into a 24-well plate. Three parallel wells were set up. The culture medium was added with EdU (C10341-1, Guangzhou RiboBio Co., Ltd., Guangzhou, Guangdong, China) until the concentration reached 10 μmol/L. After a 2-h incubation, the cells in each well were fixed with 4% paraformaldehyde in PBS at room temperature for 15 min and incubated for 20 min with 0.5% TritonX-100/PBS and with Apollo® 567 (100 μL, RiboBio) for 30 min in darkness. Next, the cells were cultured for 30 min with 100 μL 1 × Hoechst 33342 reaction solution. Finally, the positive cells were analyzed under the fluorescence microscope (FM-600, Shanghai Pudan Optical instrument Co., Ltd., Shanghai, China), under which the red stained cells were reflective of the proliferated cells. Three visual fields were arbitrarily chosen under the microscope.

### Flow cytometric analysis

The cells were subjected to a 20-min centrifugation at 3000 r/min at 48 h post-transfection and resuspended with PBS to adjust the concentration into 1 × 10^5^ cells/mL. After that, the cells were fixed at 4 °C with 1 mL precooled (− 20 °C) 75% ethanol for 1 h, centrifuged for 5 min at 1500 r/min, and water-bathed with 100 μL RNase A (Thermo Fisher Scientific) at 37 °C in darkness for 30 min. The cells were then cultured for 30 min with propidium iodide (400 μL, PI, Sigma-Aldrich Chemical Company, St. Louis, MO, USA) at 4 °C in darkness. The cell cycle observation was conducted using a flow cytometer (Beckman Coulter, Inc., Brea, CA, USA) at 488 nm red fluorescence.

The cells were trypsinized (Thermo Fisher Scientific, free of ethylene diamine tetraacetic acid) at 48 h post-transfection and centrifuged successively at 3000 r/min for 30 min and at 3000 r/min for 15 min. Following the protocols of an Annexin-V-fluorescein isothiocyanate (FITC) cell apoptosis detection kit (Sigma), Annexin-V-FITC/PI reagent was prepared with N-2-hydroxyethylpiperazine-N′-2-ethanesulfonic acid (HEPES) buffer, Annexin-V-FITC, and PI at a ratio of 50:1:2. Cells were stained at room temperature for 15 min with 100 μL staining solution and resuspended with 1 mL HEPES buffer. Lastly, cell apoptosis was evaluated using a flow cytometer at 488 nm.

### Enzyme-linked immunosorbent assay (ELISA)

The RA-FLSs in logarithmic growth phase at 48 h post-transfection were plated in a 24-well plate at 1 × 10^6^ cells each well. Following culture for 24 h, the supernatant was centrifuged at 4 °C at 1800*g*. After 1 min, the IL-1β level was measured as per the instructions of the ELISA kit (ab100704, 1:1000).

### Bioinformatics prediction

RA-related dataset GSE103578 was found in the Gene Expression Omnibus (GEO) database (https://www.ncbi.nlm.nih.gov/geo/) and analyzed with the application of the R language limma package (http://master.bioconductor.org/packages/release/bioc/html/limma.html). The differentially expressed genes were screened out on the conditions of adj.*p*.Val < 0.05 and |LogFoldchange (FC)| > 2. We then plotted a heatmap of these differentially expressed genes using the R language pheatmap package (https://cran.r-project.org/web/packages/pheatmap/index.html).

### Fluorescence in situ hybridization (FISH) for subcellular localization of PVT1

According to Ribo^TM^ lncRNA FISH probe Mix (Red) (RiboBio), after the confluence of the cells reached about 80% after 1 day of culture, the cells were then treated with 4% paraformaldehyde (1 mL) at room temperature and cultured with 2 μg/mL protease K, glycine, and phthalation reagent, successively. After prehybridization and hybridization at 42 °C for 1 h and overnight, respectively, the cells were stained with PBS containing 0.1% Tween-20-diluted 4′,6-diamidino-2-phenylindole (1:800) for 5 min. The analysis was carried out using a fluorescence microscope (Olympus, Tokyo, Japan), with images attained under five different fields.

### RNA binding protein immunoprecipitation (RIP) assay

A RIP kit (Millipore Corp, Billerica, MA, USA) was employed to assess the binding of miR-543 to PVT1 and SCUBE2. Following an ice bath with lysis buffer (P0013B, Beyotime Biotechnology Co., Ltd., Shanghai, China) for 5 min and a 10-min centrifugation at 4 °C at 14000 rpm, a portion of the supernatant was incubated with the rabbit anti-mouse antibody to IgG (ab109489, 1:100, Abcam). The remaining supernatant together with 50 μL beads were incubated at room temperature with 5 μg antibodies against Ago2 (ab32381, 1:50, Abcam) for 30 min. The aforementioned antibodies were from Abcam. After detachment with proteinase K, the RNA was extracted for subsequent PCR.

### Luciferase reporter assay

With the purpose of constructing a luciferase report vector, SCUBE2-3′untranslated region (UTR) and PVT1 containing miR-543 binding sites were inserted into pGL3 plasmids. The SCUBE2-3′UTR-mutant (MUT) fragment and the PVT1-MUT fragment of the binding site mutation were inserted into the pGL3 plasmid by the point mutation method. The inserted sequence was verified to be correct by sequencing. PGL3-lncRNA PVT1, pGL3-lncRNA PVT1-MUT, pGL3-SCUBE2-3′UTR, pGL3-SCUBE2-3′UTR-MUT recombinant vectors, and Rellina were co-transfected into 293T cells with miR-543 mimic or NC mimic. The cells were lysed 48 h after transfection, and the luciferase reporter gene was analyzed by a dual luciferase reporter gene analysis system (Promega, Madison, WI, USA) following the instructions of a Dual Luciferase Assay Kit (K801-200, BioVision, Inc., Exton, PA, USA) with the help of a fluorescence detector (Promega, Madison, WI, USA).

### Data analysis

All sample data were analyzed with Statistical Product and Service Solutions (SPSS 21.0) software (IBM Corp. Armonk, NY, USA). All data were displayed as mean ± standard deviation for three repeated individual experiments for each group. The one-way analysis of variance (ANOVA) was employed for the comparison among multi-groups, of which comparing between two groups adopted Tukey’s post hoc test. Differences were regarded as statistically significant if *p* < 0.05.

## Results

### PVT1 is highly expressed in synovial tissues of RA patients and rat models

**Fig. 1:**
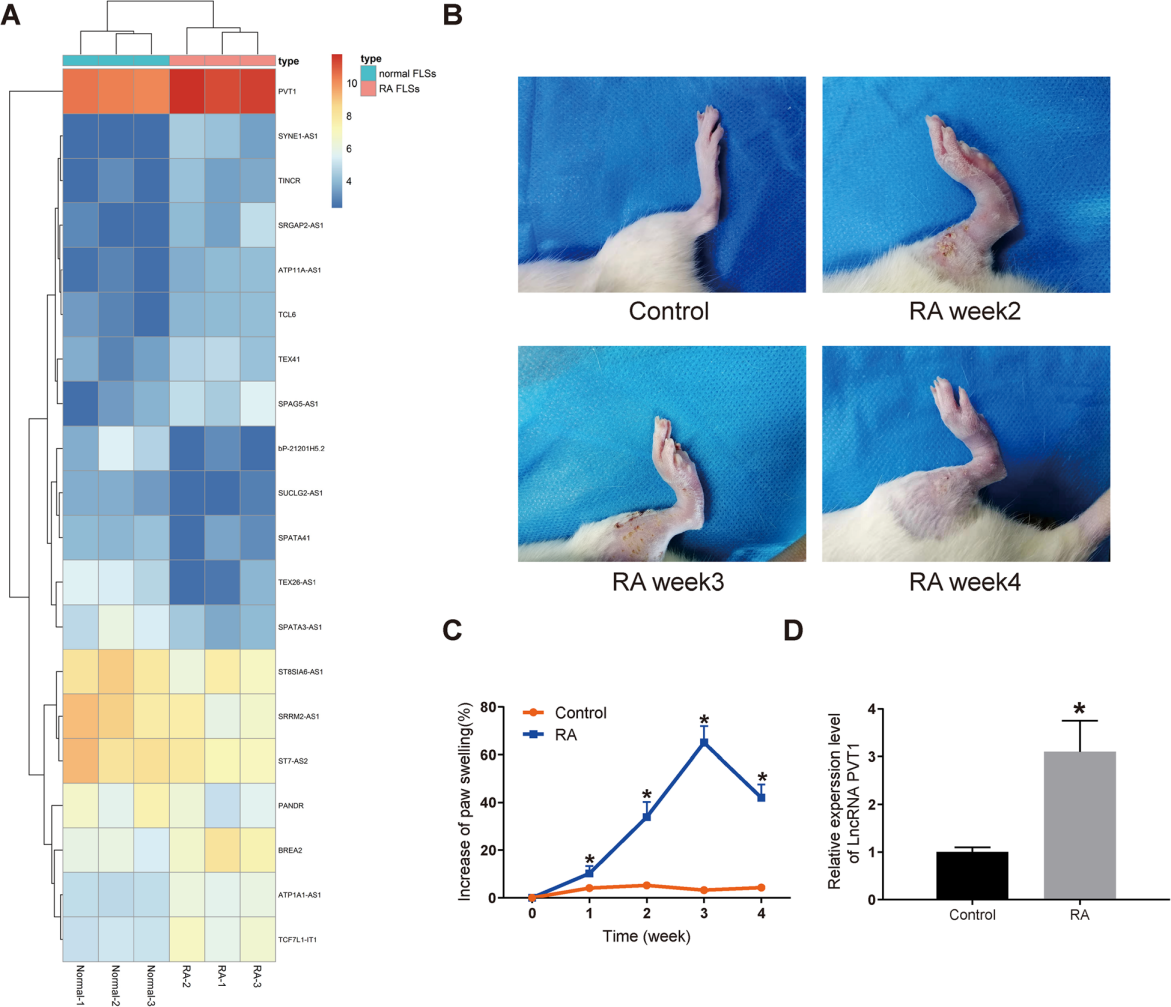
High expression of PVT1 is identified in synovial tissues of RA patients and rats. **a** The heatmap of top 30 differentially expressed lncRNAs in synovial tissues of 3 healthy people and 3 patients with RA analyzed by human lncRNA expression array V3.0 (AS-LNC-H-V4.0, Arraystar Inc.). **b** Observation of the hind paw of control and RA rats at the 2nd, 3rd, and 4th week after modeling. **c** A chart of the increase of paw swelling (%). **d** PVT1 expression in synovial tissues of RA and control rats by RT-qPCR. *p < 0.05 vs. the control rats; *n* = 7 for each group. All data were measurement data and presented as mean ± standard derivation; ANOVA was used for the comparison among multi-groups

First of all, we compared the differentially expressed lncRNAs in synovial tissues of three healthy people and three patients with RA. The differential expression of lncRNAs was analyzed by microarray. After homogenizing the original data, we screened a total of 133 differentially expressed lncRNAs using Limma Rstudio package using LogFC >1.5 and *p* value < 0.05 as screening conditions. Among them, 61 lncRNAs were downregulated and 72 lncRNAs were upregulated. The heatmap showed some differentially expressed lncRNAs (Fig. 1a), among which the difference of PVT1 expression was the most obvious.

As shown in Fig. 1b, clinical symptoms including swelling and redness of the hind paw were visible in RA rats at the 2nd week after the modeling, which were alleviated at the 4th week. The swelling degree of the hind paw of rats was analyzed. The findings revealed that in comparison with the control rats, the swelling degree of the RA rats was remarkably increased at the 1st, 2nd, 3rd, and 4th week (*p* < 0.05; Fig. 1c), indicating the successful modeling of RA rats.

After successful induction, the synovial tissues from control and RA rats were extracted to conduct RT-qPCR for the PVT1 expression measurement. The results exhibited that the PVT1 expression in the RA rats was markedly increased versus that in the control rats (*p* < 0.05; Fig. 1d). These data obtained imply that the upregulation of PVT1 in the synovial tissues may be associated with the development of RA.

### PVT1 knockdown inhibits the proliferation of FLSs and the release of IL-1β in RA and promotes their apoptosis

**Fig. 2:**
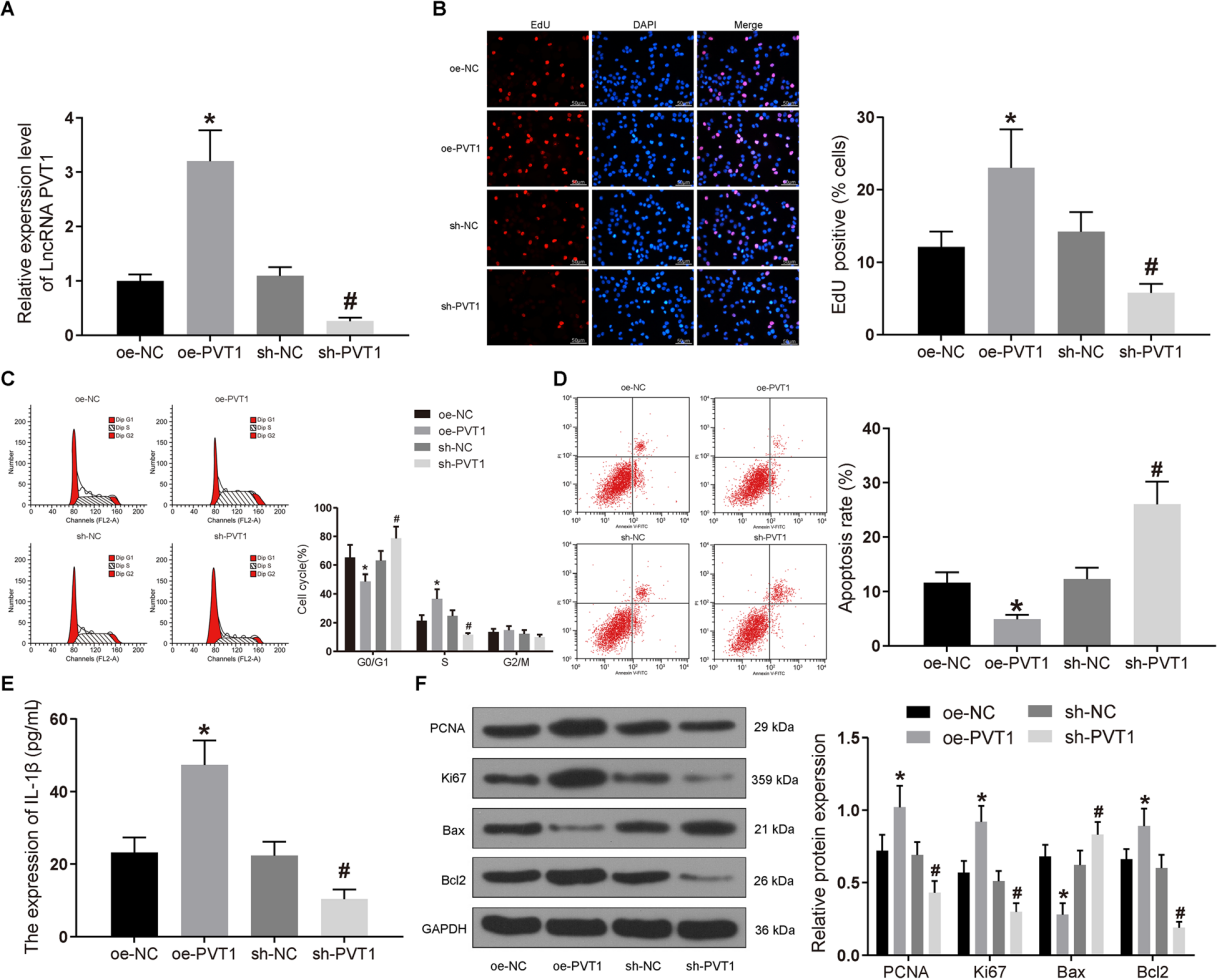
The proliferation and inflammation of synovial cells are halted, and apoptosis is stimulated by the knockdown of PVT1. FLSs were treated with oe-PVT1 or sh-PVT1 with oe-NC or sh-NC as controls. **a** PVT1 expression in cells of each group determined by RT-qPCR. **b** The proliferation of cells in each group detected by EdU staining (× 100). **c** Cell cycle changes in each group measured by flow cytometry. **d** The apoptosis rate of cells in each group detected by flow cytometry. **e** The expression of IL-1β in cell culture media determined by ELISA. **f** The expression of Bax, Bcl2, PCNA, and Ki67 in cells tested by western blot analysis. *p < 0.05 vs*.* synovial cells treated with oe-NC; ^#^*p* < 0.05 vs. synovial cells treated with sh-NC. All data were measurement data and presented as mean ± standard derivation. The ANOVA was used for the comparison among multi-groups, of which comparing between two groups adopted Tukey’s post hoc test; the experimental data was obtained from the average of 3 independent experiments

Based on the results of microarray analysis and previous experiments, we learned that PVT1 was upregulated in synovial tissues of RA, and then RA-FLSs were transfected with oe-PVT1 or sh-PVT1. The transfection efficacy was then examined by RT-qPCR, suggesting that PVT1 expression was reduced following sh-PVT1 treatment (*p* < 0.05), whereas promoted expression was identified after oe-PVT1 delivery (*p* < 0.05; Fig. 2a).

At that time, the cell proliferation and apoptosis were examined by EdU staining and flow cytometry. Figure 2b–d reveals that the proliferation rate of oe-PVT1-treated cells was drastically elevated, the proportion of cells in G0/G1 phase was diminished, and the proportion of cells in S phase was enhanced, accompanied by reduced apoptosis rate (*p* < 0.05). Furthermore, sh-PVT1 transfection contributed to significantly decreased proliferation and the proportion of S, while it contributed to increased proportion of G0/G1 and apoptosis rate (*p* < 0.05). The release of IL-1β in culture medium was measured by ELISA. The results exhibited that (Fig. 2e) the IL-1β secretion was significantly promoted by oe-PVT1 (*p* < 0.05), while it was significantly diminished by sh-PVT1 (*p* < 0.05). Finally, the expression patterns of apoptosis-associated proteins Bax and Bcl2 as well as proliferation-related proteins PCNA and Ki67 were assessed by western blot assays. PCNA, Ki67, and Bcl2 in oe-PVT1-treated cells were significantly upregulated, whereas Bax expression was decreased (*p* < 0.05). Conversely, PCNA, Ki67, and Bcl2 were diminished due to sh-PVT1 treatment, while Bax was remarkably increased (*p* < 0.05; Fig. 2f). Collectively, sh-PVT1 has the potency to suppress the proliferation and IL-1β secretion of RA-FLSs and to induce its apoptosis.

### SCUBE2 is overexpressed and miR-543 is downregulated in synovial tissues of RA patients and rats

**Fig. 3:**
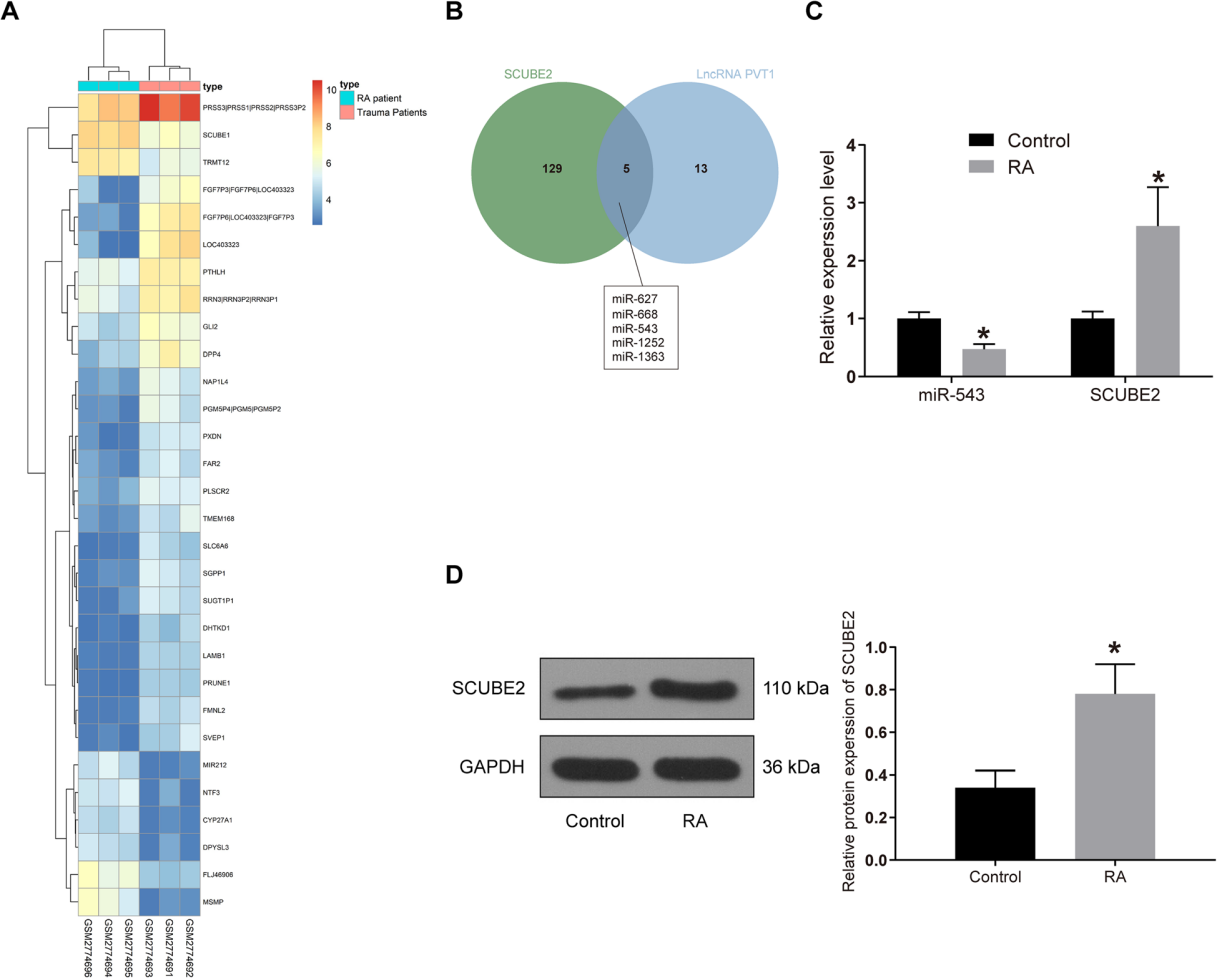
High expression of SCUBE2 and low expression of miR-543 are corroborated in synovial tissues of RA rats. **a** The heatmap of top 30 differentially expressed mRNAs in synovial tissues. **b** Prediction and screening of binding miRNAs of PVT1 and SCUBE by StarBase. **c** miR-543 expression and mRNA expression of SCUBE2 in synovial tissues of control rats and RA rats measured by RT-qPCR. **d** The protein expression of SCUBE2 in synovial tissues of control rats and RA rats examined by western blot analysis; *p < 0.05 vs. the control rats; *n* = 7 for each group. All data were measurement data and presented as mean ± standard derivation; ANOVA was used for the comparison among multi-groups

Subsequently, we analyzed the GSE103578 dataset in the GEO database, which included FLSs from trauma patients and RA patients. A total of 190 differentially expressed mRNAs were screened by LogFC >2, *p* value < 0.05 using the Limma Rstudio package, of which 108 mRNAs were downregulated and 92 mRNAs were upregulated. Figure 3a is the heatmap exhibiting some differentially expressed mRNAs. Moreover, we found that silencing of SCUBE2 could slow down the injury of RA. Therefore, we chose SCUBE2 as our target.

In order to further determine the ceRNA mechanism, we predicted the miRNAs that shared binding relationships with both SCUBE2 and PVT1 by the StarBase website. A total of five miRNAs, miR-627, miR-668, miR-543, miR-1252, and miR-1363 were screened out (Fig. 3b). Among them, miR-543 has been reported to inhibit the expression of tumor necrosis factor superfamily member 15, thus attenuating inflammatory reaction and cell apoptosis [13], and to reduce the neuroinflammatory response by blocking the NF-κB pathway [17].

miR-543 and SCUBE2 expression in the synovial tissues of control and RA rats were assessed using RT-qPCR and western blot assays. The findings illustrated that miR-543 expression in RA rats decreased significantly relative to control rats, while SCUBE2 expression increased significantly at both mRNA and protein levels (*p* < 0.05; Fig. 3c, d). Hence, the overexpression of SCUBE2 and downregulation of miR-543 in the synovial tissues may be linked to the RA development.

### PVT1 binds to miR-543 to upregulate SCUBE2 expression

**Fig. 4:**
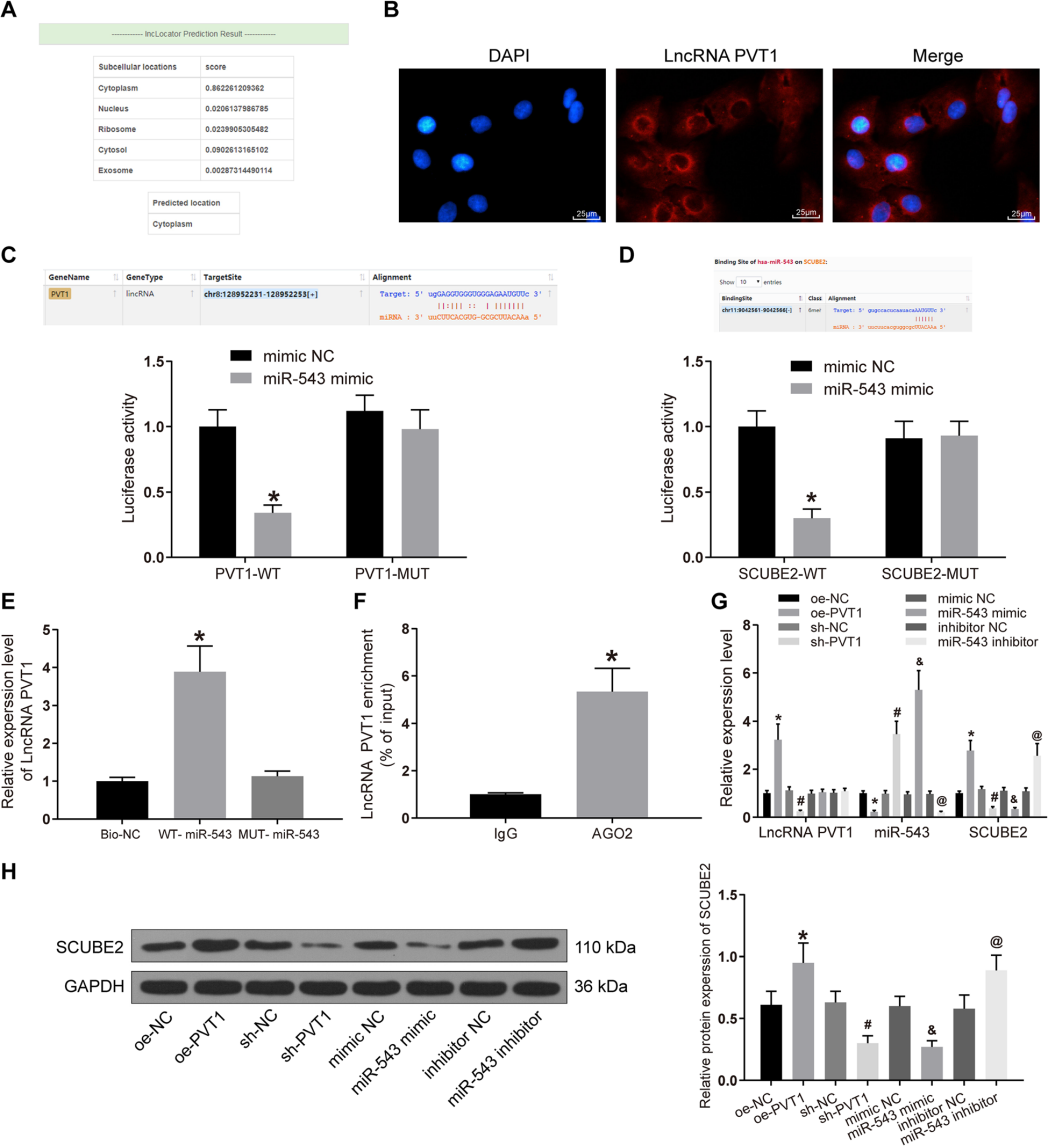
PVT1 interacts with miR-543 in RA-FLSs to promote SCUBE2 expression. **a** The PVT1 localization in cytoplasm predicted by LncLocator. **b** Localization of PVT1 in FLSs of RA by FISH (× 100). **c** The binding relationship between PVT1 and miR-543 verified by dual luciferase reporter gene assay, *p < 0.05 vs. the mimic NC group. **d** The binding relationship between SCUBE2 and miR-543 verified by dual luciferase reporter gene assay, *p < 0.05 vs. the mimic NC group. **e** The binding relationship between PVT1 and miR-543 verified by the RNA pull-down assay, *p < 0.05 vs. the Bio-NC group. **f** PVT1 is enriched by AGO2 verified by RIP, *p < 0.05 vs. the IgG group. **g** PVT1 and miR-543 expression and the mRNA expression of SCUBE2 in each group was detected by RT-qPCR, *p < 0.05 vs. the oe-NC group, ^#^*p* < 0.05 vs. the sh-NC group, ^&^*p* < 0.05 vs. the mimic NC group, ^@^*p* < 0.05 vs. the inhibitor NC group. **h** The protein expression of SCUBE2 in each group was detected by western blot analysis, *p < 0.05 vs. the oe-NC group, ^#^*p* < 0.05 vs. the sh-NC group, ^&^*p* < 0.05 vs. the mimic NC group, ^@^*p* < 0.05 vs. the inhibitor NC group. All data were measurement data and presented as mean ± standard derivation. The ANOVA was used for the comparison among multi-groups, of which comparing between two groups adopted Tukey’s post hoc test; the experimental data was obtained from the average of 3 independent experiments

We predicted that PVT1 was principally located in the cytoplasm as revealed by Lncatlas database, which was further substantiated by our FISH assay. The blue staining stood for the nucleus and the red signified PVT1 (Fig. 4a), demonstrating that PVT1 was primarily expressed in the cytoplasm. It was suggested that PVT1 may function by the ceRNA mechanism.

The binding relationships between miR-543 and SCUBE2 and between miR-543 and PVT1 (Fig. 4b, c) were substantiated by the luciferase reporter assay. As expected, miR-543 mimic profoundly inhibited the luciferase activity of PVT1 wild type (WT) relative to mimic NC (*p* < 0.05), rather than the luciferase activity of PVT1-MUT (*p* > 0.05). Also, miR-543 mimic repressed the luciferase activity of SCUBE2-WT similarly. The above results showed that PVT1 may bind to miR-543 to interact with the SCUBE gene.

Whether PVT1 could bind to miR-543 directly was validated by RNA pull-down. The results showed that (Fig. 4d) PVT1 was pulled-down more by WT-miR-543 than MUT-miR-543 and Bio-NC (*p* < 0.05), implying a direct binding relationship between PVT1 and miR-543. RIP assay was performed to confirm whether PVT1 directly interacted with AGO2 in RA-FLSs, which displayed that (Fig. 4e) PVT1 was enriched by antibody to AGO2 in RA-FLSs. As a result, PVT1 could form complexes with AGO2.

PVT1, miR-543, and SCUBE2 expression patterns were examined by the means of RT-qPCR and western blot assays after overexpression or silencing of PVT1 and miR-543 in RA-FLSs, respectively (Fig. 4f, g). The data provided that PVT1 and SCUBE2 expression was increased significantly and that of miR-543 decreased after overexpression of PVT1 (*p* < 0.05). After silencing of PVT1, the opposite trends were observed (*p* < 0.05). Moreover, we conducted the same experiments in RA-FLSs overexpressing miR-543, there was no remarkable change in the PVT1 expression, but miR-543 expression was significantly upregulated, while SCUBE2 was remarkably diminished at both mRNA and protein levels (*p* < 0.05). Meanwhile, we observed the opposite trends after the downregulation of miR-543 (Fig. 4h). In conclusion, PVT1 is participated in the proliferation, apoptosis, and IL-1β secretion of RA-FLSs by sponging miR-543 to upregulate SCUBE2.

### Silencing miR-543 promotes the proliferation and IL-1β secretion of RA-FLSs and inhibits its apoptosis

**Fig. 5:**
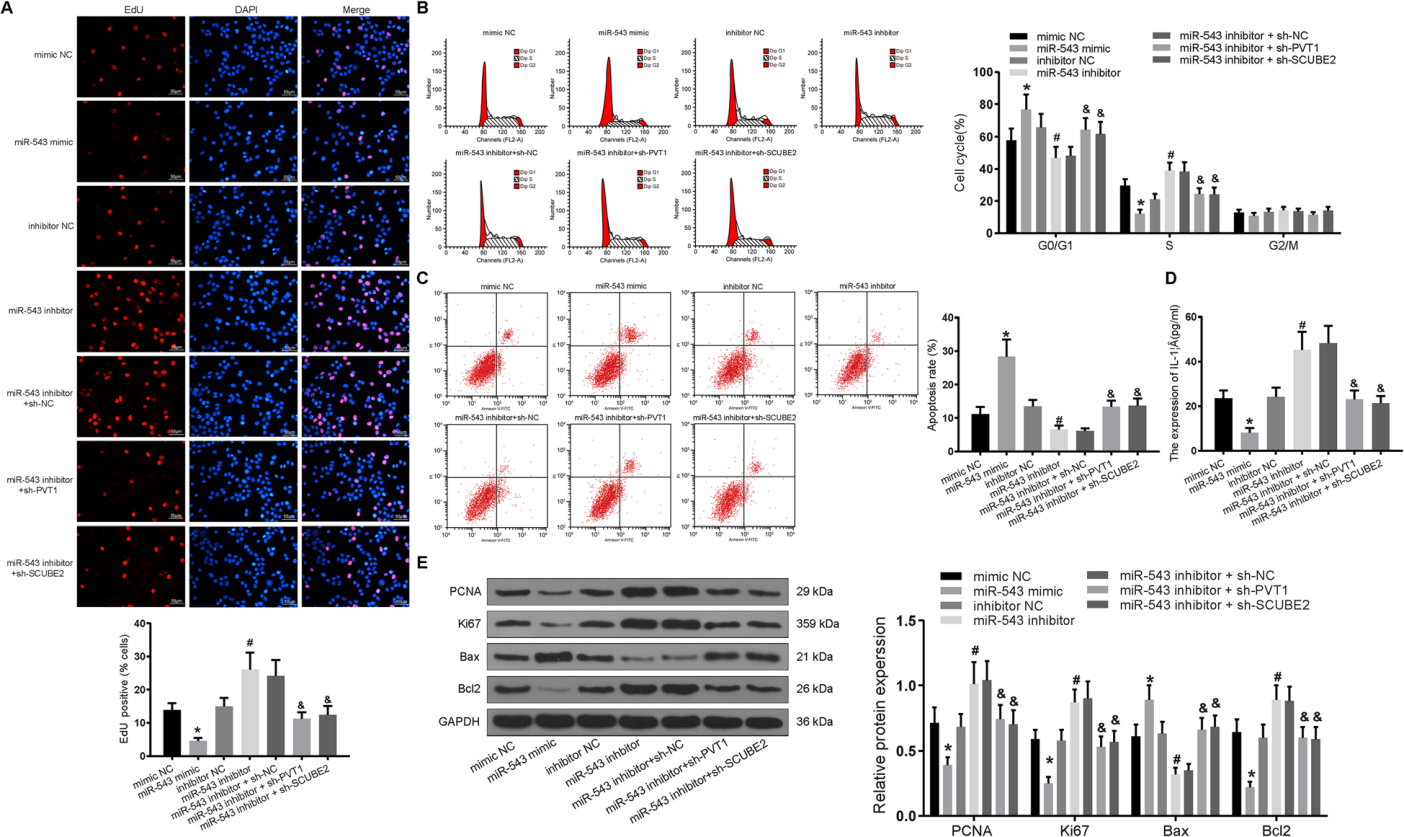
Silencing of miR-543 promotes RA-FLS proliferation and inflammation and inhibits apoptosis, which could be reversed by silencing of PVT1 or SCUBE2 in RA. Synovial cells were treated with sh-SCUBE2 or sh-PVT1 in the presence of miR-543 inhibitor or miR-543 mimic alone. **a** The proliferation of cells in each group detected by EdU staining (× 100). **b** Cell cycle changes in each group measured by flow cytometry. **c** The apoptosis rate of cells in each group detected by flow cytometry. **d** The expression of IL-1β in cell culture media determined by ELISA. **e** Expression of Bax, Bcl2, PCNA, and Ki67 in cells tested by western blot analysis. *p < 0.05 vs. synovial cells treated with mimic NC; ^#^*p* < 0.05 vs. synovial cells treated with inhibitor NC; ^&^*p* < 0.05 vs. synovial cells treated with miR-543 inhibitor + sh-NC. All data were measurement data and presented as mean ± standard derivation. The ANOVA was used for the comparison among multi-groups, of which comparing between two groups adopted Tukey’s post hoc test; the experimental data was obtained from the average of 3 independent experiments

To further observe the mechanism where miR-543 governs RA-FLSs in RA, these cells were co-transfected with sh-SCUBE2 or sh-PVT1 and miR-543 inhibitor or miR-543 mimic alone. The cell viability, cell cycle, and cell apoptosis rate were detected by EdU and flow cytometry. As displayed by Fig. 5a–c, the cell proliferation and S phase ratio were significantly reduced, while G0/G1 phase ratio and apoptosis rate were significantly promoted by miR-543 mimic (*p* < 0.05). Meanwhile, miR-543 inhibitor exerted a stimulative role in cell proliferation and S phase ratio, while it exerted a repressive role in G0/G1 phase ratio and apoptosis rate. Rescue experiments exhibited that silencing of SCUBE2 or PVT1 reversed the function of miR-543 inhibitor in cell viability and apoptosis.

The release of IL-1β in cell culture medium was measured by ELISA. Figure 5d illustrates that the secretion of IL-1β following the miR-543 mimic treatment was significantly diminished, and the secretion of IL-1β after miR-543 inhibitor treatment was remarkably restored (*p* < 0.05). Compared with the miR-543 inhibitor + sh-NC treatment, the secretion of IL-1β was significantly decreased following sh-SCUBE2 + miR-543 inhibitor and sh-PVT1 + miR-543 inhibitor treatments (*p* < 0.05).

As revealed by western blot analysis (Fig. 5e), relative to the mimic NC delivery, the PCNA, Ki67, and Bcl2 expression was decreased, while Bax expression was enhanced by the miR-543 mimic delivery (*p* < 0.05). Conversely, the protein expression of PCNA, Ki67, and Bcl2 after miR-543 inhibitor treatment was markedly promoted, while the protein expression of Bax was reduced (*p* < 0.05). The PCNA, Ki67, and Bcl2 expression was profoundly decreased, while the Bax expression was significantly reduced after the miR-543 inhibitor + sh-SCUBE2 and miR-543 inhibitor + sh-PVT1 treatments (*p* < 0.05). Taken together, the facilitated RA-FLS proliferation and inflammation as well as hindered apoptosis induced by miR-543 inhibition could be counteracted by silencing of PVT1 or SCUBE2.

## Discussion

First, we found in the present study that PVT1 was highly expressed in synovial tissues of RA patients by microarray analysis. Similarly, we found that PVT1 expression was elevated in synovial tissues of RA rats. Then, we performed the gain- and loss-of-function experiments of PVT1 in FLSs and found that overexpression of PVT1 significantly promoted the proliferation of FLSs and inflammation, yet inhibited apoptosis as evidenced by boosted Ki67, PCNA, IL-1β, and Bcl-2 expression, whereas it hampered Bax expression. We further noted that PVT1 bound to miR-543, and miR-543 targeted SCUBE2 by bioinformatics prediction. Moreover, further miR-543 inhibitor in the presence of silencing SCUBE2 or PVT1 could promote FLS proliferation and IL-1β secretion, while inhibiting apoptosis.

Most epidemiologic records suggest that the incidence of RA is about 0.5–1.0%, and around 70 to 80% of RA patients have autoantibodies, indicating that RA represents itself as an autoimmune disease [18]. Considerable efforts to define the epigenome of RA have concentrated on FLS of the synovial intimal lining that invade the cartilage, demonstrating an exceptional aggressive phenotype in RA patients [19]. During this study, we were set to examine the functions of PVT1 in RA-FLSs to further clarify the specific mechanisms linked to the pathogenesis of RA. PVT1 is located at chromosome 8q24, a well-acknowledged risk locus for cancer and was promoted in bladder cancer tissue and linked to advanced histological grades and higher tumor stage in addition to lymph node metastases [20]. Furthermore, PVT1 was explored to significantly decrease miR-365 expression in Huh7 and HepG2 cells through sponging effect through the luciferase reporter assay [21]. During the past decade, the ceRNA theory involving lncRNA-miRNA-mRNA network has been established in the progression of various kinds of disorders, including autoimmune diseases [22]. For instance, a novel lncRNA GAPLINC has been monitored to encourage tumor-like behaviors of RA-FLS through binding with miR-382-5p and miR-575 [23]. A loop of has-circ-0028198/has-circ-0092317/XIST/miR-543/aspartate beta-hydroxylase or has-circ-0028198/has-circ-0092317/XIST/miR-543/phosphodiesterase 3B has been implicated in oxidized, low-density, lipoprotein-stimulated foam cells in atherosclerosis [24]. Furthermore, miR-543 was significantly reduced under senescence-inducing conditions and could modulate the aging process of human mesenchymal stem cells [25]. Meanwhile, miR-543-5p expression in the rat spinal cord was diminished after spinal cord injury [17]. SCUBE2 has been reported to regulate the development of vertebrae by regulating the expression of bone morphogenetic protein [26]. Also, the anti-rheumatic role of SCUBE2 through regulating synovial angiogenesis has been reported previously [14].

The important function of PCNA, a main player of the cell cycle modulation, on proliferation and migration of FLSs has been previously investigated [27]. In line with our findings, the anti-apoptotic protein Bcl2 was enhanced, whereas the pro-apoptotic gene Bax was suppressed by silencing of signal transducer and activator of transcription 3 through delivering small interfering RNA in RA-FLSs [28]. Interestingly, PVT1 could directly bind to miR-149 as an endogenous sponge RNA and miR-149 suppression reversed the protective roles of PVT1 knockdown in attenuating IL-1β-evoked inflammation in osteoarthritic chondrocytes [29]. In addition, silencing of PVT1 promoted cell proliferation, yet suppressed inflammation in C28/I2 cells stimulated by IL-1β, which was counteracted by the deficiency of its target miR-27b-3p [30]. The contributory roles of sh-PVT1 in the elevation of RA-FLS apoptosis and suppression of proliferation and IL-1β release was achieved by competitively binding to miR-543 to downregulate SCUBE2 expression. Thus, we demonstrated in detail the effects of the PVT1/miR-543/SCUBE2 axis on the regulation of RA-FLS proliferation and apoptosis, which is a great advantage of our research. However, we did not further investigate the effect of the signaling pathway downstream of the PVT1/miR-543/SCUBE2 axis on RA. For example, NF-κB signaling plays an important role in the pathogenesis and progression of RA [31]. Besides, miR-145-5p affects RA by regulating Wnt1/β-catenin signaling [32]. In the next step, we will study the effect of signaling downstream of PVT1/miR-543/SCUBE2 on RA.

## Conclusion

**Fig. 6:**
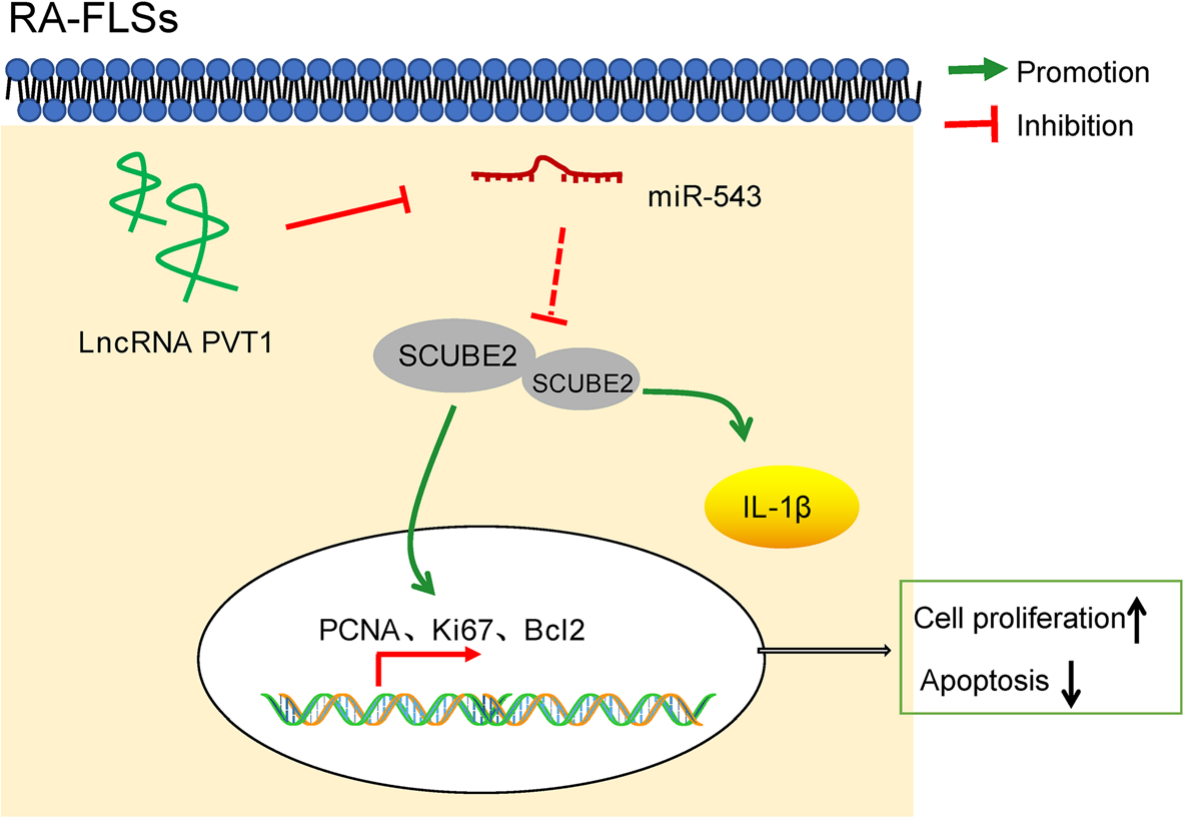
In RA-FLSs, upregulated PVT1 enhanced SCUBE2 expression by sponging miR-543, thereby facilitating cell proliferation and suppressing apoptosis via the enhancement of PCNA, Ki67, and Bcl2 expression, as well as promoting the production of IL-1β

In aggregate, the current study indicates that downregulation of PVT1 reduces proliferation and IL-1β release, while it induces apoptosis of RA-FLS by mediating the miR-543/SCUBE2 axis (Fig. 6). However, we cannot exclude the involvement of other signaling pathways in the regulation of inflammation due to the complex microenvironments, which would be the focus of our future researches.

## Data Availability

All the data generated or analyzed during this study are included in this published article.

## Abbreviations

RA: Rheumatoid arthritis
lncRNAs: Long non-coding RNAs
FLSs: Fibroblast-like synoviocytes
ceRNA: Competitive endogenous RNA
PVT1: Plasmacytoma variant translocation 1
miR-543: MicroRNA-543
SCUBE2: Signal peptide-CUB-EGF-like containing protein 2
IL-1β: Interleukin-1β
GEO: Gene Expression Omnibus
FC: Foldchange
FISH: Fluorescence in situ hybridization
MUT: Mutant
UTR: Untranslated region
PBS: Phosphate-buffered saline
oe: Overexpression
sh: Short hairpin RNA
NC: Negative control
RT-qPCR: Reverse transcription-quantitative polymerase chain reaction
cDNA: Complementary deoxyribonucleic acid
GAPDH: Glyceraldehyde-3-phosphate dehydrogenase
PCNA: Proliferating cell nuclear antigen
Bax: Bcl-2-associated X protein
EdU: 5-Ethynyl-2′-deoxyuridine
PI: Propidium iodide
FITC: Fluorescein isothiocyanate
HEPES: N-2-Hydroxyethylpiperazine-N′-2-ethanesulfonic acid
ANOVA: One-way analysis of variance

## References

[1] Smolen JS, Aletaha D, Barton A, Burmester GR, Emery P, Firestein GS, Kavanaugh A, McInnes IB, Solomon DH, Strand V, Yamamoto K (2018). Rheumatoid arthritis. Nat Rev Dis Primers..

[2] Aletaha D, Smolen JS (2018). Diagnosis and management of rheumatoid arthritis: a review. JAMA..

[3] Bartok B, Firestein GS (2010). Fibroblast-like synoviocytes: key effector cells in rheumatoid arthritis. Immunol Rev..

[4] de Oliveira PG, Farinon M, Sanchez-Lopez E, Miyamoto S, Guma M (2019). Fibroblast-like synoviocytes glucose metabolism as a therapeutic target in rheumatoid arthritis. Front Immunol..

[5] Bi X, Guo XH, Mo BY, Wang ML, Luo XQ, Chen YX, Liu F, Olsen N, Pan YF, Zheng SG (2019). LncRNA PICSAR promotes cell proliferation, migration and invasion of fibroblast-like synoviocytes by sponging miRNA-4701-5p in rheumatoid arthritis. EBioMedicine..

[6] Fang Y, Tu J, Han D, Guo Y, Hong W, Wei W (2020). The effects of long non-coding ribonucleic acids on various cellular components in rheumatoid arthritis. Rheumatology (Oxford)..

[7] Li H, Chen S, Liu J, Guo X, Xiang X, Dong T, Ran P, Li Q, Zhu B, Zhang X, Wang D, Xiao C, Zheng S (2018). Long non-coding RNA PVT1-5 promotes cell proliferation by regulating miR-126/SLC7A5 axis in lung cancer. Biochem Biophys Res Commun..

[8] Wang C, Zhu X, Pu C, Song X (2018). Upregulated plasmacytoma variant translocation 1 promotes cell proliferation, invasion and metastasis in colorectal cancer. Mol Med Rep..

[9] Yang Q, Yu Y, Sun Z, Pan Y (2018). Long non-coding RNA PVT1 promotes cell proliferation and invasion through regulating miR-133a in ovarian cancer. Biomed Pharmacother..

[10] Zhang CW, Wu X, Liu D, Zhou W, Tan W, Fang YX, Zhang Y, Liu YQ, Li GQ (2019). Long non-coding RNA PVT1 knockdown suppresses fibroblast-like synoviocyte inflammation and induces apoptosis in rheumatoid arthritis through demethylation of sirt6. J Biol Eng..

[11] Hur K, Kim SH, Kim JM (2019). Potential implications of long noncoding RNAs in autoimmune diseases. Immune Netw..

[12] Jiang H, Ma R, Zou S, Wang Y, Li Z, Li W (2017). Reconstruction and analysis of the lncRNA-miRNA-mRNA network based on competitive endogenous RNA reveal functional lncRNAs in rheumatoid arthritis. Mol Biosyst..

[13] Li XZ, Lv CL, Shi JG, Zhang CX. MiR-543-3p promotes locomotor function recovery after spinal cord injury by inhibiting the expression of tumor necrosis factor superfamily member 15 in rats. Eur Rev Med Pharmacol Sci. 2019;23:2701-2709. doi:10.26355/eurrev_201904_17540. 10.26355/eurrev_201904_17540 31002119

[14] Yang M, Guo M, Hu Y, Jiang Y (2013). Scube regulates synovial angiogenesis-related signaling. Med Hypotheses..

[15] Jinesh GG, Flores ER, Brohl AS (2018). Chromosome 19 miRNA cluster and CEBPB expression specifically mark and potentially drive triple negative breast cancers. PLoS One..

[16] Livak KJ, Schmittgen TD (2001). Analysis of relative gene expression data using real-time quantitative PCR and the 2(-delta delta C(T)) method. Methods..

[17] Zhao CL, Cui HA, Zhang XR. MiR-543-5p inhibits inflammation and promotes nerve regeneration through inactivation of the NF-kappaB in rats after spinal cord injury. Eur Rev Med Pharmacol Sci. 2019;23:39-46. doi:10.26355/eurrev_201908_18626. 10.26355/eurrev_201908_18626 31389572

[18] Okada Y, Eyre S, Suzuki A, Kochi Y, Yamamoto K (2019). Genetics of rheumatoid arthritis: 2018 status. Ann Rheum Dis..

[19] Ai R, Laragione T, Hammaker D, Boyle DL, Wildberg A, Maeshima K, Palescandolo E, Krishna V, Pocalyko D, Whitaker JW, Bai Y, Nagpal S, Bachman KE, Ainsworth RI, Wang M, Ding B, Gulko PS, Wang W, Firestein GS (2018). Comprehensive epigenetic landscape of rheumatoid arthritis fibroblast-like synoviocytes. Nat Commun..

[20] Yu C, Longfei L, Long W, Feng Z, Chen J, Chao L, Peihua L, Xiongbing Z, Hequn C (2019). LncRNA PVT1 regulates VEGFC through inhibiting miR-128 in bladder cancer cells. J Cell Physiol..

[21] Yang L, Peng X, Jin H, Liu J (2019). Long non-coding RNA PVT1 promotes autophagy as ceRNA to target ATG3 by sponging microRNA-365 in hepatocellular carcinoma. Gene..

[22] Yan S, Wang P, Wang J, Yang J, Lu H, Jin C, Cheng M, Xu D (2019). Long non-coding RNA HIX003209 promotes inflammation by sponging miR-6089 via TLR4/NF-kappaB signaling pathway in rheumatoid arthritis. Front Immunol..

[23] Mo BY, Guo XH, Yang MR, Liu F, Bi X, Liu Y, Fang LK, Luo XQ, Wang J, Bellanti JA, Pan YF, Zheng SG (2018). Long non-coding RNA GAPLINC promotes tumor-like biologic behaviors of fibroblast-like synoviocytes as microRNA sponging in rheumatoid arthritis patients. Front Immunol..

[24] Wang L, Zheng Z, Feng X, Zang X, Ding W, Wu F, Zhao Q (2019). circRNA/lncRNA-miRNA-mRNA network in oxidized, low-density, lipoprotein-induced foam cells. DNA Cell Biol..

[25] Lee S, Yu KR, Ryu YS, Oh YS, Hong IS, Kim HS, Lee JY, Kim S, Seo KW, Kang KS. miR-543 and miR-590-3p regulate human mesenchymal stem cell aging via direct targeting of AIMP3/p18. Age (Dordr). 2014;36:9724. doi:10.1007/s11357-014-9724-2. 10.1007/s11357-014-9724-2 PMC4259092 25465621

[26] Xavier GM, Cobourne MT (2011). Scube2 expression extends beyond the central nervous system during mouse development. J Mol Histol..

[27] Jia W, Wu W, Yang D, Xiao C, Su Z, Huang Z, Li Z, Qin M, Huang M, Liu S, Long F, Mao J, Liu X, Zhu YZ (2018). Histone demethylase JMJD3 regulates fibroblast-like synoviocyte-mediated proliferation and joint destruction in rheumatoid arthritis. FASEB J..

[28] Sun X, Han Y, Liu Y, Tang Y, Wang J (2019). Proliferation and apoptosis of rheumatoid arthritis fibroblast-like synoviocytes following signal transducer and activator of transcription 3 RNA interference delivery. J Cell Biochem..

[29] Zhao Y, Zhao J, Guo X, She J, Liu Y. Long non-coding RNA PVT1, a molecular sponge for miR-149, contributes aberrant metabolic dysfunction and inflammation in IL-1beta-simulated osteoarthritic chondrocytes. Biosci Rep. 2018;38. 10.1042/BSR20180576. 10.1042/BSR20180576 PMC6165834 30126849

[30] Lu X, Yu Y, Yin F, Yang C, Li B, Lin J, Yu H (2019). Knockdown of PVT1 inhibits IL-1beta-induced injury in chondrocytes by regulating miR-27b-3p/TRAF3 axis. Int Immunopharmacol..

[31] Carvalho AMS, Heimfarth L, Pereira EWM, Oliveira FS, Menezes IRA, Coutinho HDM, Picot L, Antoniolli AR, Quintans JSS, Quintans-Junior LJ. Phytol, a chlorophyll component, produces antihyperalgesic, anti-inflammatory, and antiarthritic effects: possible NFkappaB pathway involvement and reduced levels of the proinflammatory cytokines TNF-alpha and IL-6. J Nat Prod. 2020. 10.1021/acs.jnatprod.9b01116. 10.1021/acs.jnatprod.9b01116 32091204

[32] Dinesh P, Kalaiselvan S, Sujitha S, Rasool M (2020). MiR-145-5p mitigates dysregulated Wnt1/beta-catenin signaling pathway in rheumatoid arthritis. Int Immunopharmacol..

